# A robust dilute-and-inject ion chromatography - Tandem mass spectrometry for urinary glyphosate quantification

**DOI:** 10.1016/j.mex.2026.104001

**Published:** 2026-06-09

**Authors:** Mari-Vorgan Louyer, Cécile Jabalot, Arthur David, Fabien Mercier

**Affiliations:** a Univ Rennes, Inserm, EHESP, Irset (Institut de Recherche en Santé, Environnement et Travail) - UMR_S 1085, Rennes F-35000, France; b France Exposome - Breizh Exposome Leres, French National Infrastructure in Exposomics, EIRENE Node, Rennes, France

**Keywords:** Human biomonitoring, IC-MS/MS, Environmental health, Biomarker of exposure, Herbicide

## Abstract

Glyphosate is one of the most widely used herbicides in the world, whose potential effects on human health are still the subject of debate today. To assess the associated risks, reliable biomonitoring data are required using validated robust and sensitive analytical methods. In this context, an ion chromatography coupled with tandem mass spectrometry (IC-MS/MS) method was developed to quantify glyphosate in human urine. The method is based on direct injection of diluted urine samples. A labeled internal standard (^13^C_2_^15^N_1_ glyphosate) is added prior to injection to correct for losses or matrix effects during the analysis. The method limit of quantification (LOQ) is 0.1 ng/mL in urine. The working range was validated between 0.1 and 20 ng/mL. Method trueness was assessed at three concentration levels (LOQ, 2 and 20 ng/mL), and a full validation of the method was performed to characterize the analytical performance in terms of precision and measurement uncertainty.

• Direct injection ion chromatography coupled with tandem mass spectrometry
• Validation of the method according to international standards

## Graphical abstract

**Figure:**
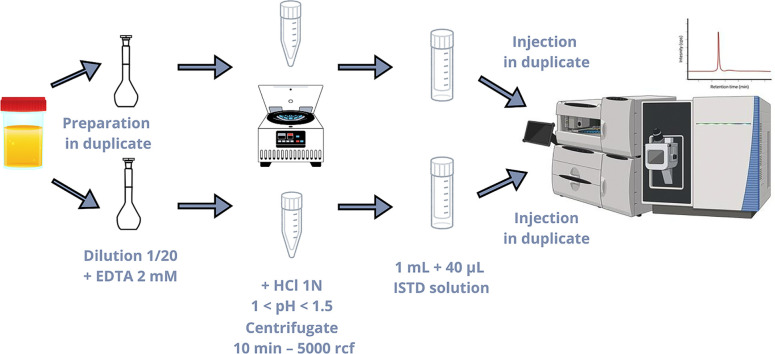

Specifications table

**Table:** 

**Subject area**	Environmental Science
**More specific subject area**	Analytical chemistry
**Name of your method**	Determination of glyphosate in human urine by IC-MS/MS
**Name and reference of original method**	None
**Resource availability**	None

## Background

Despite glyphosate being one of the most widely used herbicides globally, biomonitoring data remain uncertain due to analytical challenges related to its physicochemical properties [1]. Indeed, available studies show considerable heterogenicity, in terms of exposure levels, analytical methods and quantification limits, highlighting the lack of robust and sensitive standardized validated methods for the affordable measurement of glyphosate in urine samples [2]. Ion chromatography coupled with tandem mass spectrometry (IC-MS/MS) methods are increasingly used for polar compounds on water samples [3] and more recently on animal or human biological samples [[4], [5], [6]]. This technique presents several advantages compared with conventional methods such as liquid or gas chromatography coupled with tandem mass spectrometry (LC-MS/MS or GC–MS/MS) following derivatization and solid phase extraction (SPE) [[7], [8], [9]]. These advantages include direct sample injection with minimal or no pretreatment, the elimination of a derivatization step, and sufficient sensitivity achieved without sample concentration and even when the sample is diluted. In this context, we propose an IC-MS/MS method to analyze glyphosate in urine samples, whose performance was assessed in terms of linearity, precision, uncertainty and reproducibility according to international standards.

## Method details

### Reagents and chemicals

ISO 17034 compliant certified standards for calibration and controls were purchased from VWR International (Radnor, PA, USA) and Cluzeau info Labo (Sainte-Foy-La-Grande, France) respectively. The isotopically labeled internal standard (^13^C_2_^15^N_1_ glyphosate) was purchased from LGC Labor GmbH (Augsburg, Germany). Individual stock solutions (1 g/L) were prepared in ultra-pure water generated by a Milli-Q Advantage A10 system by Millipore (Burlington, Massachusetts, USA) and Evian water (Danone, France) was used for standard preparation and sample dilution.

Hydrochloric acid (HCl) 1 N and synthetic matrix (Sigmatrix) urine diluent for matrix procedural controls were purchased from Sigma-Aldrich (Saint-Louis, MO, USA). EDTA (ethylenediaminetetraacetic acid disodium salt dihydrate) was purchased from Chem-Lab (Zedelgem, Belgium).

Pooled urine samples for method validation were obtained by donation with consents.

### Preparation of urine samples

Each sample was prepared in duplicate. The urine sample was homogenized and 20-fold diluted with Evian water and EDTA (2 mM) in class A volumetric flask. 2 mL of the diluted sample were transferred into an Eppendorf tube and adjusted to pH 1 to 1.5 with pH indicator strips using HCl 1 N. After centrifugation at 5000 rcf for 10 min with Eppendorf 5804R centrifuge (Hamburg, Germany), 1 mL of the supernatant was transferred into a vial containing 40 μL of the internal standard solution (^13^C_2_^15^N_1_ glyphosate) corresponding to a final concentration of 1.2 ng/mL and then mixed. Each duplicate of each sample was injected twice in IC-MS/MS. Stability of samples at −20 °C has been validated for 30 months (cf. Method validation and research article).

### Ion chromatography

The separation step was performed using a Thermo Scientific™ Dionex™ ICS-6000. Fifty microliters of the diluted sample were injected with an AS-DV Autosampler maintained at 10 °C. Separation was conducted on an IonPac™ AS31-IC column (250 × 2 mm) kept at 30 °C during the analysis with a flow rate of 0.3 mL/min. An electrolytic eluent generator using a KOH supply (EGC 500 KOH cartridge) generated ultrapure hydroxide eluent to perform the separation. The gradient was run with a hydroxide concentration of 17 mM at the beginning held for 18 min, increased to 85 mM in 11 min, held for 11 min, then returned to the initial mobile phase composition (17 mM of hydroxide), and held for 5 min. The total run time was 50 min. The system included a Continuously Regenerated Anion Trap Column for carbonate removal of the eluent. To minimize the background conductivity of the eluent, a Thermo Dionex anion electrolytically regenerated suppressor (AERSe, 2 mm) was installed before the conductivity detector with an external pump for suppressor regeneration or detection in the MS spectrometer, delivering a flow at 0.4 mL/min. After the conductivity detector, a diverter valve guided the flow to the mass spectrometer or to waste. Programming the collection window between 23 and 29 min avoided salts from urine samples clogging the ion source. System control and data processing were performed by Chromeleon 7.3 software (Thermo Scientific, Sunnyvale, CA).

### Mass spectrometry

Two precursor/product ion transitions were monitored for native and labeled glyphosate. The target ion transition with the highest intensity was used for quantitation, while the second target ion transition was used for confirmation. The IC-MS/MS analyses were performed in negative heated electrospray ionization (H-ESI) mode. The mass spectrometer ion source parameters were 400 °C and 3200 V for the ion transfer tube temperature and the spray voltage, respectively. The sheath gas and auxiliary gas were set to 50 and 10 (Thermo arbitrary units), respectively. The collision energies (V) were optimized for all diagnostic transitions of native and labeled glyphosate and are summarized in Table 1. Argon was used as the collision gas.

**Table 1:** Optimized detection parameters for native and labeled glyphosate.

Compound	Molecular weight (g/mol)	Transition	RF Lens (V)	Collision energy (V)	MRM transition
Glyphosate	169	Quantitation	39	24	168 > 63
		Confirmation	39	10	168 > 150
^13^C_2_^15^N_1_ Glyphosate	172	Quantitation	59	24	171 > 63
		Confirmation	59	11	171 > 153

### Calibration

The nine calibration samples were prepared on the day of use in Evian water according to Table 2 from a 100 ng/mL glyphosate stock solution. Evian water was selected instead of ultrapure water in order to introduce mineral content into the calibration standards. Hydrochloric acid was added to each standard to achieve a pH of 1.5 and an internal standard solution was added prior to injection.

**Table 2:** Concentrations of calibration samples (LOQ: limit of quantification).

Level number	Concentration of glyphosate in the calibration samples (ng/mL)	Corresponding concentration in urine samples (ng/mL)
1 (LOQ)	0.0050	0.10
2	0.010	0.20
3	0.025	0.50
4	0.050	1.0
5	0.10	2.0
6	0.25	5.0
7	0.50	10
8	1.0	20
9	2.0	40

The calibration curve was generated using a linear regression with an internal standard and a 1/amount weighting. This weighting reduces the influence of higher concentration levels, resulting in an approximately uniform contribution of both low and high concentrations to the regression.

### Quality control

The internal quality control procedure for each batch was carried out with the following analysis:

− calibration blank sample (Evian water);
− instrumental blank sample (no injection);
− procedural blank sample (synthetic urine matrix);
− instrumental control samples at 0.005 ng/mL (LOQ) and 0.1 ng/mL prepared in Evian water; These controls are analyzed at least every 20 injections in each batch.
− matrix procedural QC samples at 2 levels: LOQ level (0.1 ng/mL) and medium level (2 ng/mL), prepared in a synthetic urine matrix following the entire analytical process.

### Result validation

Glyphosate was identified by comparing retention times and the abundance of the internal standard peak areas between calibration and urine samples. For positive samples, the confirmation criteria for relative abundances were set to 20–150% of the calibration samples as defined by the laboratory and the confirmation criteria for relative retention times were set to 97.5–102.5% of the calibration samples, as defined by the international standard [10]. The signal-to-noise ratio (S/N) of the quantitation peak had to be greater than 10.

The data validation protocol included several conditions: i) the determination coefficient of the calibration curve had to be greater than 0.99, ii) the concentration of a substance measured in the calibration samples had to be within ±40% of its theoretical concentration value at the LOQ level and ±20% at all other levels, iii) the response of a substance (ISTD response ratio) in the calibration, instrumental and procedural blank samples had to be lower than 50% of that in the procedural calibration sample at the LOQ level, iv) the concentration of a substance measured in the matrix instrumental control samples had to be within ±60% of its theoretical concentration value at the LOQ level and ±30% at the intermediate level, v) the concentration of a substance measured in the matrix procedural QC samples prepared from pooled urine samples had to be within ±60% of its theoretical concentration value at the LOQ level and ±30% at the intermediate level and vii) the concentration of a substance measured in the urine samples had to be within the method working range.

For each urine sample, the final result was the average of the four measurements obtained from the two injections of each preparation duplicate. The coefficient of variation (CV) was calculated from the four results obtained (two injections of each preparation). If this CV exceeds 25%, one of the four results may be excluded. The result was validated if the difference between at least three out of four measurements was under 25%. Otherwise, reinjection of the sample and/or new preparation was performed if possible. If these conditions were not met, results were not validated, and samples were reanalysed if possible.

## Method validation

Performance of this method was evaluated according to AFNOR standard NF T90–210 [11] and internal procedures that comply with the requirements of the French Committee for Accreditation (Cofrac).

Linearity was evaluated using 5 calibrations in 5 different batches by calculating the coefficient of determination (r^2^). For each of the nine calibration samples, the deviation between the measured concentration and the corresponding true concentration was calculated and expressed as a percentage. This deviation was obtained by subtracting the true concentration from the measured concentration, dividing the result by the true concentration, and multiplying by 100.

For accuracy, precision and uncertainty, 6 different real urine samples spiked at 3 concentration levels (0.1 (LOQ), 2 and 20 ng/mL) and analyzed in duplicate in 6 different batches were used. The 6 real urine samples used for this test were representative of the range of urine samples to be analyzed using this method in terms of urinary density, pH and osmolality (Table 3). This enables the evaluation of matrix effects across the entire application range of the method.

**Table 3:** Characterization of the 6 real urine samples used for method validation.

Urine	pH	Osmolality (mOsm/kg)	Specific gravity (unitless)
A	5.5	853.5	1.0232
B	6.0	387.0	1.0112
D	6.5	551.5	1.0176
E	6.2	764.5	1.0205
F	6.2	729.5	1.0184
G	7.0	260.0	1.0069

Accuracy (%) was determined by dividing the mean concentration obtained from the duplicates in each batch by the corresponding theoretical concentration and then multiplying the result by 100. The theoretical concentration represented the difference between the spiked and non-spiked levels.

Precision (within-lab-reproducibility) in % was calculated by dividing a combination of repeatability standard deviation and reproducibility standard deviation by the average of the concentration.

Uncertainty is a combination of trueness and reproducibility and was calculated with a confidence level of 95%.

The results of the method validation are presented in Table 4.

**Table 4:** Results of the method validation.

Test	Results		
Linearity Calibration	r^2^ > 0.99 Deviation < 20% for each calibration level		
Level (ng/mL)	0.1	2.0	20
Accuracy (%)	109%	106%	101%
Precision (%)	18	8.0	5.0
Uncertainty (%)	42	22	12

Long term stability at −20 °C was assessed using four real urine samples (A–D). Samples were spiked at 2 ng/mL and analysed in triplicate at Day 0. Aliquots were then stored at −20 °C and reanalysed at months 2, 3, 6, 12, 14, 19 and 30, with duplicate analyses performed at each time point. Glyphosate stability was evaluated by calculating the ratio of concentrations measured at each time point relative to the mean concentration measured at Day 0. Results were visualized using control charts, with a maximum allowable deviation (MAD) of ±20%, as defined by the laboratory.

The laboratory also successfully participates every year in proficiency testing programs for glyphosate in urine: the OSEQAS (Organic Substances in urine Quality Assessment Scheme) program organized by the QUEBEC Toxicology Centre/INSPQ and the German external quality assessment scheme (G-EQUAS). The results obtained by the laboratory are presented in Table SI.1.

Finally, the laboratory obtained Cofrac (French Committee for Accreditation) accreditation for this method, in accordance with the ISO/CEI 17,025 standard (accreditation no 8–3557, https://www.cofrac.fr).

## Limitations

Although the method developed for urinary glyphosate determination provides satisfactory performance for routine biomonitoring, it still presents several limitations that must be acknowledged. First, the analytical conditions used in this study do not allow the simultaneous quantification of AMPA, the main glyphosate environmental metabolite [12] and glufosinate, another non-selective phosphorus containing amino-acid type herbicide. Although AMPA is frequently measured alongside glyphosate, its relevance as a biomarker of human exposure has been questioned, notably because it may originate from environmental degradation rather than direct exposure pathways [13]. As for glufosinate, it was already measured in urine samples, but not quantified [14,15]. In addition, the chromatographic run time remains relatively long (50 min), substantially higher than that of conventional LC-MS/MS methods, and the analysis of replicates may represent a limitation for high-throughput applications. Another constraint is the current availability of the analytical platform: at this stage, only a single supplier provides an integrated solution compatible with this approach, which may hinder broader adoption and limit flexibility in instrument configuration.

Despite these limitations, several avenues offer promising perspectives for overcoming them. The development of ultra-high-pressure ion chromatography (UHPIC) systems, the emergence of new stationary phases specifically optimized for highly polar compounds, and the potential application of high-resolution mass spectrometry (HRMS) may lead to a significant reduction of run times while improving selectivity and sensitivity. Likewise, advances in sample preparation strategies, including on-line clean-up or derivatization-free workflows, could simplify the analytical process and open the way toward multiplexed determination of glyphosate and AMPA within a single method. These developments collectively represent promising directions for future work aimed at increasing throughput, robustness, and analytical coverage.

## Ethics statements

Pooled urine samples for method validation were obtained by donation with consents.

## CRediT authorship contribution statement

**Mari-Vorgan Louyer:** Investigation, Validation, Formal analysis, Writing – original draft, Writing – review & editing, Visualization. **Cécile Jabalot:** Validation, Formal analysis, Investigation, Writing – review & editing. **Arthur David:** Conceptualization, Writing – review & editing, Supervision. **Fabien Mercier:** Conceptualization, Writing – review & editing, Supervision, Project administration.

## Declaration of competing interest

The authors declare that they have no known competing financial interests or personal relationships that could have appeared to influence the work reported in this paper.

## Data Availability

Data will be made available on request.

## Appendix Supplementary materials

Image, application 1
