# Supplementary material for: A robust dilute-and-inject ion chromatography - Tandem mass spectrometry for urinary glyphosate quantification
Source: MethodsX. 2026 Jun 9;17:104001. doi: 10.1016/j.mex.2026.104001 (PMC13276513; doi:10.1016/j.mex.2026.104001)
Supplement: Supplementary file 1 [file mmc1.docx]

Table SI.1. Interlaboratory results

| **Trial** | **Date** | **Sample**  **ID** | **Reference value**  **(ng/mL)** | **Laboratory result**  **(ng/mL)** | **z-score** | **Trueness (%)** |
| --- | --- | --- | --- | --- | --- | --- |
| **INSPQ_OSEQAS** | **06/05/2022** | **OS-U-E2201** | 0.645 | **0.81** | **1.28** | 26 |
|  | **06/05/2022** | **OS-U-E2202** | 0.673 | **0.67** | **-0.02** | 0 |
|  | **06/05/2022** | **OS-U-E2203** | 0.542 | **0.78** | **2.20** | 44 |
|  | **03/05/2023** | **OS-U-E2301** | 0.727 | **0.83** | **0.71** | 14 |
|  | **03/05/2023** | **OS-U-E2302** | 3.99 | **3.78** | **-0.26** | -5 |
|  | **03/05/2023** | **OS-U-E2303** | 2.03 | **2.15** | **0.30** | 6 |
|  | **13/09/2023** | **OS-U-E2304** | 3.89 | **3.70** | **-0.24** | -5 |
|  | **13/09/2023** | **OS-U-E2305** | 3.35 | **3.30** | **-0.07** | -1 |
|  | **13/09/2023** | **OS-U-E2306** | 1.73 | **1.80** | **0.20** | 4 |
| **G-EQUAS** | **29/09/2023** | **RV-72-9A** | **0.13** | **0.13** | **0.0** | 0 |
|  | **29/09/2023** | **RV-72-9B** | **0.77** | **0.80** | **-0.43** | 4 |
|  | **16/09/2024** | **RV-74-**9A | **0.26** | **0.24** | **-0.31** | -8 |
|  | **16/09/2024** | **RV-74-9B** | **1.38** | **1.10** | **-0.81** | -20 |
|  | **24/03/2025** | **RV-75-**9A | 0.31 | **0.33** | **0.26** | 6 |
|  | **24/03/2025** | **RV-75-**9B | 0.84 | **0.86** | **0.10** | 2 |
|  | 26/09/2025 | **RV-76-**9A | 0.16 | **0.15** | **-1.00** | -6 |
|  | 26/09/2025 | **RV-76-**9B | 1.98 | **2.20** | **1.57** | 11 |
